# RNA: packaged and protected by VLPs†

**DOI:** 10.1039/c8ra02084a

**Published:** 2018-06-12

**Authors:** Po-Yu Fang, Jessica C. Bowman, Lizzette M. Gómez Ramos, Chiaolong Hsiao, Loren Dean Williams

**Affiliations:** School of Chemistry and Biochemistry, Georgia Institute of Technology Atlanta GA 30332 USA loren.williams@chemistry.gatech.edu; School of Chemical and Biomolecular Engineering, Georgia Institute of Technology Atlanta GA 30332 USA; Institute of Biochemical Sciences, National Taiwan University Taipei 10617 Taiwan Republic of China

## Abstract

Virus Like Particles (VLPs) are devices for RNA packaging, protection and delivery, with utility in fundamental research, drug discovery, and disease treatment. Using *E. coli* for combined expression and packaging of non-viral RNAs into Qβ VLPs, we investigated the extent of chemical protection conferred by packaging of RNA in VLPs. We also probed relationships between packaging efficiency and RNA size, sequence and intrinsic compaction. We observe that VLP packaging protects RNA against assault by small diffusible damaging agents such as hydroxyl radicals and divalent cations. By contrast, the extent of unmediated cleavage, in the absence of reactive species, is the same for RNA that is free or packaged within VLPs, and is very slow. *In vivo* packaging of RNA within VLPs appears to be more efficient for intrinsically compact RNAs, such as rRNA, and less efficient for unstructured, elongated RNA such as mRNA. Packaging efficiency is reduced by addition of the ribosome binding site to a target RNA. The Qβ hairpin is necessary but not sufficient for efficient packaging.

## Abstract 2

VLP packaging is most efficient for compact RNA, and protects RNA against assault by small diffusible damaging agents.

## Graphical abstract

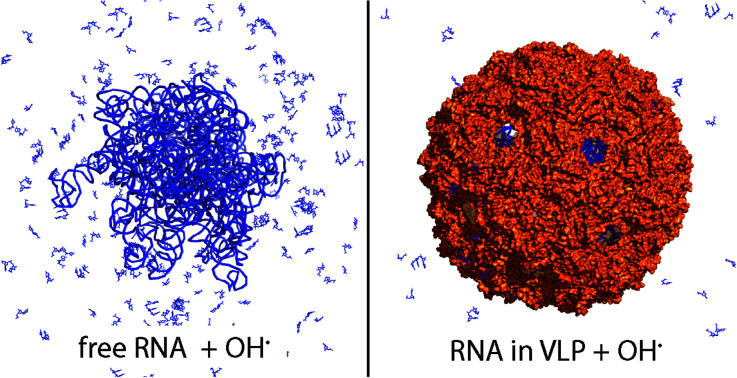

## Introduction

RNA-based tools have utility in fundamental research, drug discovery, and disease treatment^1–3^ in part because of the programmable specificity of base-pairing interactions. Small interfering RNAs (siRNAs) and microRNAs (miRNAs) modulate gene expression by inhibiting translation or by stimulating degradation of mRNAs.^4,5^ RNA can be active as antibacterial or antiviral agents.^6,7^

Impediments to broadly applicable RNA-based therapeutics remain. Cellular uptake is inhibited by the high anionic charge of RNA.^8^ Chemical and biological stability of RNA is low.^9,10^ RNA production on large scales by *in vitro* transcription^11^ or solid-phase synthesis^12^ is problematic.^13,14^

The packaging of RNA by protein(s) *in vivo*, in coupled RNA production and packaging systems, may be effective for overcoming at least a subset of these challenges.^15^ RNA expression *in vivo*^16–18^ can employ recombinant nucleic acids that encode polymerase promoters, affinity tags, functional ‘target’ sequences and genes for packaging proteins.

Virus Like Particles (VLPs) are packaging systems that can be used to deliver RNA and other cargo. Qβ and MS2 VLPs are derived from single-strand RNA bacteriophages. Their genomes are small and their protein capsids are *T* = 3 icosahedral nanoparticles with diameters of ∼30 nm, formed of 180 copies of the coat protein (CP).^19–21^ Qβ VLPs are hollow with pores 1.4 nm in diameter.^20^

**Fig. 1:**
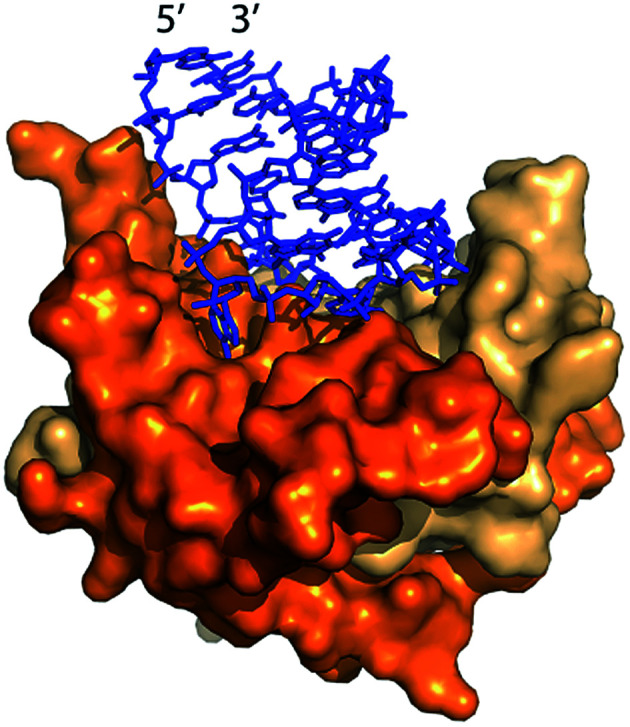
The Qβ hairpin (blue) in complex with the coat protein (two copies are shown) in the assembled Qβ VLP (PDB 4L8H).^25^

Lau *et al.*^22^ and Fang *et al.*^15^ previously demonstrated packaging of non-viral RNA in Qβ VLPs by co-expressing target RNA and CP from distinct plasmids in *E. coli*. *In vivo* packaging of target RNA in VLPs is promoted by an RNA hairpin (hp), derived from Qβ RNA, which has high affinity for CP (Fig. 1).^23,24^ CP binds to hp during VLP assembly *in vivo* through RNA backbone, sugar (2′ hydroxyl), and sequence-specific interactions.^25^ RNA secondary structure also promotes viral assembly; RNA stem-loops are thought to promote capsid formation, while CP interactions with RNA promote RNA collapse.^26,27^

Biological stability of RNA can be increased by fusing target RNA to stable folds such as 5S rRNA or tRNA. These fusions allow simultaneous production and purification of active RNA in fermentation-based systems.^28–33^ tRNA fusions were produced and packaged in MS2 VLPs within *E. coli* by Ponchon *et al.*^14^ We recently demonstrated simultaneous production and packaging of active RNAi in Qβ VLPs using a novel scaffold.^15^

Here, we explore factors that influence the stability and quantity of target RNA that is packaged in Qβ VLPs *in vivo*. We report that packaging in VLPs chemically protects RNA from small diffusible chemicals that can readily penetrate the VLP. It appears that packaging chemically stabilizes RNA by mechanisms beyond direct exclusion of reactive species from proximity to the RNA. We show that intrinsic compaction of RNA may increase the efficiency of assembly. Intrinsically compact RNA, here derived from rRNA, appears to package with high efficiency *in vivo*.

## Methods

### Qβ CP and Qβ VLP–RNA expression vectors

**Fig. 2:**
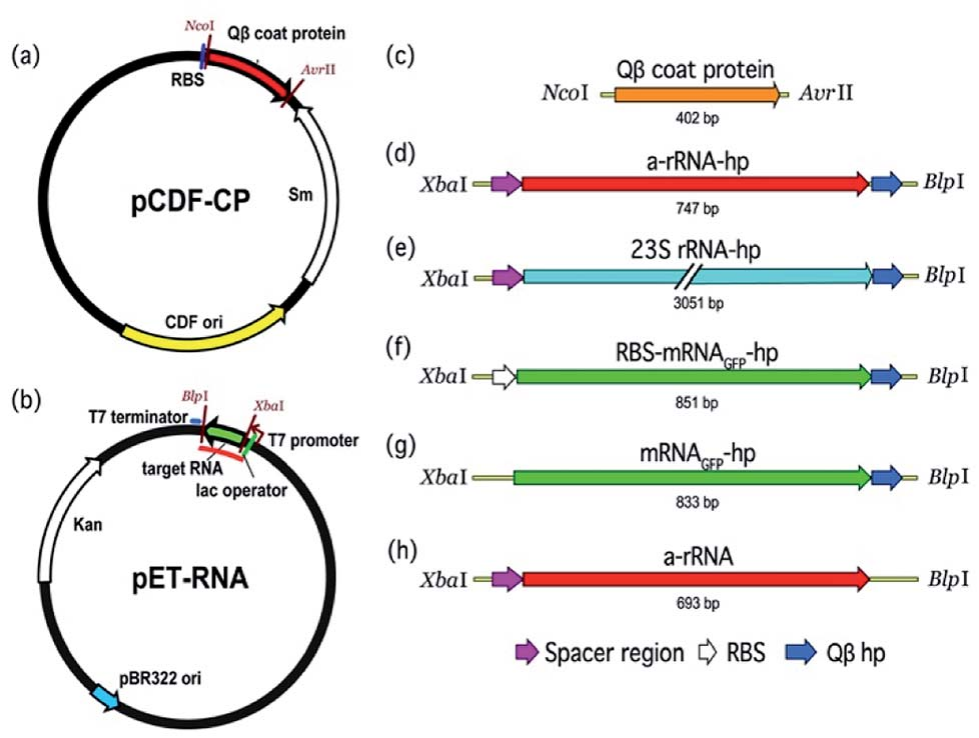
The pCDF-CP and pET-RNA plasmids used to co-express VLP protein and RNA in *E. coli*. (a) The protein expression vector (pCDF-CP) encoding Qβ VLP. (b) The generic RNA production vector (pET-RNA) used to express the series of RNAs in this study. The red line on pET-RNA plasmid (b) indicates the RNA transcript. (c) The gene cloned to pCDF-1b to create pCDF-CP is Qβ coat protein. (d–h) Genes cloned to pET-28b (+) to create the series of pET-RNAs are (d) a-rRNA-hp, (e) 23S rRNA-hp, (f) RBS-mRNA_GFP_-hp, (g) mRNA_GFP_-hp, and (h) a-rRNA. ‘hp’ is Qβ hairpin; ‘RBS’ is ribosomal binding site.

A two-plasmid expression system was used for production of Qβ VLPs and non-viral RNA in *E. coli* (Fig. 2). Two origins of replication and two antibiotic resistance markers were used within the same host. The pBR322 origin of replication and kanamycin resistance gene of pET-28b (+) (Novagen) are compatible with the CloDF13 origin and streptomycin resistance gene in pCDF-1b (Novagen), allowing for co-expression in the same bacterial cell. Therefore, we chose pCDF-1b as the coat protein expression vector (pCDF-CP, Fig. 2a) and pET-28b (+) as the RNA expression vector (pET-RNA, Fig. 2b). The non-viral RNAs and Qβ CP were cloned downstream of the T7 promoter/lac operator.

#### Qβ coat protein expression vector

DNA encoding Qβ CP (NCBI reference sequence: NC_001890.1) with flanking restriction sites Nco I and Avr II was synthesized by recursive-PCR (R-PCR)^34^ (Table S1†) and cloned into pCDF-1b to generate plasmid pCDF-CP (Fig. 2). *E. coli* BL21(DE3) transformants were selected based on streptomycin resistance (50 μg mL^−1^) and screened for inserts by colony PCR.

#### RNA expression vectors

Template DNAs flanked by XbaI and BlpI restriction sites were synthesized by R-PCR^34^ (Table S2†) and cloned into pET-28b (+) to generate the pET-RNA expression vectors (Fig. 2). *E. coli* BL21(DE3) pCDF-CP/pET-RNA transformants were selected based on LB streptomycin/kanamycin resistance (50 μg mL^−1^).

### Expression of Qβ VLP and Qβ VLP–RNA

*E. coli* BL21(DE3) containing pCDF-CP vector was inoculated in NZY medium (1.0% select peptone (NZ amine), 0.5% sodium chloride, 0.5% yeast extract) containing streptomycin (50 μg mL^−1^) and incubated overnight at 37 °C. Qβ VLP production was initiated with the addition of 1% overnight culture to ZYM-5052 auto-induction medium.^35^ Production culture was incubated for 24 h at 37 °C. Cells were collected by centrifugation at 4 °C and 6500 × *g* for 30 min. Cells were resuspended in an equal volume of Qβ buffer (10 mM MgCl_2_ and 20 mM Tris–HCl, pH 7.5) and lysed by sonication at 40 watts for 3 min with 10 s on/off intervals. The lysate was centrifuged for 30 minutes at 23 400 × *g*. Ammonium sulfate precipitation (2 M) was followed by 30 minute centrifugation at 23 400 × *g* to obtain crude VLPs, which were suspended in 1 mL Qβ buffer and extracted three times with 1 : 1 *n*-butanol : chloroform. VLPs in the aqueous layer were purified by step sucrose gradient ultracentrifugation (10–40% w/v) at 40 000 rpm for 2 hours. Qβ VLPs were precipitated with 20% w/v PEG8000. Resuspended Qβ VLPs were dialyzed against dialysis buffer (20 mM Tris–HCl, pH 7.5 and 50 mM NaCl) for 2 h. Qβ VLPs were characterized by SDS polyacrylamide gel electrophoresis. Qβ VLP–RNA complexes were obtained as described above but by co-expression of pCDF-CP/pET-RNA selected with streptomycin/kanamycin (50 μg mL^−1^).

### RNA extraction from Qβ VLPs

To extract RNA from purified VLPs, 50 μg of VLP was added to extraction buffer (5% SDS, 25 mM DTT, 20 mM Tris–HCl, pH 7.5, and 50 mM NaCl) to 250 μL. The solution was incubated at room temperature for 20 minutes. An equivalent volume of low pH phenol–chloroform was then added to the solution. RNA extracts were centrifuged for 15 minutes at 16 300 × *g*. The RNA retained in the aqueous phase was precipitated in ammonium acetate at −20 °C for 24 h followed by 15 minute centrifugation at 16 300 × *g*. The RNA pellet was washed three times with 80% ethanol. RNA was dissolved in nuclease free water and stored at −80 °C. In this extraction method, RNA yield is about 4–5 μg for every 50 μg VLP or VLP–RNA. RNA samples were analyzed by denaturing urea polyacrylamide gel electrophoresis or Agilent 2100 Bioanalyzer.

### Transmission electron microscopy

TEM images were obtained with a Hitachi H-7500 electron microscope. Samples were prepared by fixing with 250 μL of glutaraldehyde (8%) per 500 μL of sample (1 μg mL^−1^). A sample volume of 15 μL was deposited on a copper grid and incubated for 5 min. Excess sample was wicked off with filter paper. The grid was then placed face down on a droplet of 2% ammonium molybdate stain for 2 min. Excess stain was wicked off with filter paper and the grid was dried for 2 h before imaging.

### Hydroxyl radical cleavage

Following established methods,^36^ VLP–RNA assemblies (250 μg) or unpackaged RNA (25 μg) were added to pre-cleavage solution (0.3% H_2_O_2_, 3.3 mM sodium ascorbate, and 20 mM Tris–HCl, pH 7.4) to a volume of 50 μL. Cleavage reactions were initiated by addition of 10 μL of freshly prepared 200 μM iron(ii) EDTA with a 2 : 1 ratio of (NH_4_)_2_Fe(SO_4_)_2_·6H_2_O to EDTA. Reactions were quenched at various timepoints (0–180 minutes) by addition of 20 μL of 0.1 M thiourea. For reactions with varying concentrations of hydroxyl radical, 10 μL of iron(ii) EDTA (0–1500 μM) was prepared and added to solutions of VLP–RNA complexes or unpackaged RNA in solution as described above. RNA extraction was performed according to the procedures above.

### Magnesium-mediated in-line cleavage

Following established methods,^37^ VLP–RNA assemblies (250 μg) or unpackaged RNA (25 μg) were added to Mg^2+^ cleavage solution (25 mM MgCl_2_, 20 mM Tris–HCl, pH 7.4) to a final volume of 60 μL. Samples were incubated at 37 °C for various time periods (0–120 hours). VLP–RNA complexes or unpackaged RNA were added to solutions of varying concentration of MgCl_2_ (0–300 mM). Samples were incubated at 37 °C for 15 minutes. Reactions were quenched by freezing at −80 °C. RNA extraction was as described above.

### Unmediated cleavage

To investigate chemical stability of the RNA in the absence of reactive species, VLP–RNA complexes or unpackaged RNAs were incubated in a solution lacking divalent cations (20 mM Tris–HCl, pH 7.4). These samples were incubated at room temperature for various time periods.

### RNA quantitation

Reaction products were run on denaturing urea polyacrylamide gels and stained with SYBR® Green II. Band intensities were integrated with AlphaView® software for FluorChem systems. Packaging efficiencies were determined by normalization of packaged target RNA to co-packaged CP mRNA from the same co-expression/purification.

## Results

Here we assessed: (i) the chemical stability conferred to RNA by packaging in Qβ VLPs, and (ii) the effect of specific RNA characteristics on *in vivo* packaging efficiency. A series of RNAs were co-expressed with Qβ VLP CP in *E. coli* (Fig. 2). The RNAs are of different lengths, extents of compaction and sequence. We investigated the chemical robustness of packaged RNA in the presence of reactive small molecules, and the effect of RNA length and structure on packaging efficiency.

### Qβ VLP packaging protects RNA from chemical degradation

To investigate the effect of VLP packaging on the chemical robustness of RNA, VLPs containing ‘a-rRNA’^38^ were constructed and treated with reactive chemicals. a-rRNA is a well-characterized RNA derived from the ancestral core of the *Thermus thermophilus* large ribosomal subunit rRNA.^38^ Here, a-rRNA was fused to the Qβ hairpin (a-rRNA-hp, Fig. 2d).

It was previously shown that VLPs protect RNA from degradation by protein nucleases,^39^ which are larger than the pore size of the VLPs. Here we investigated the ability of RNA packaged in Qβ VLPs to resist degradation by small diffusible molecules that are smaller than the VLP pore size. We compared rates of chemical degradation of a-rRNA-hp packaged in VLPs to a-rRNA-hp free in solution. The amount of uncleaved RNA was determined for a variety of timepoints.

#### Cleavage with Fenton chemistry

**Fig. 3:**
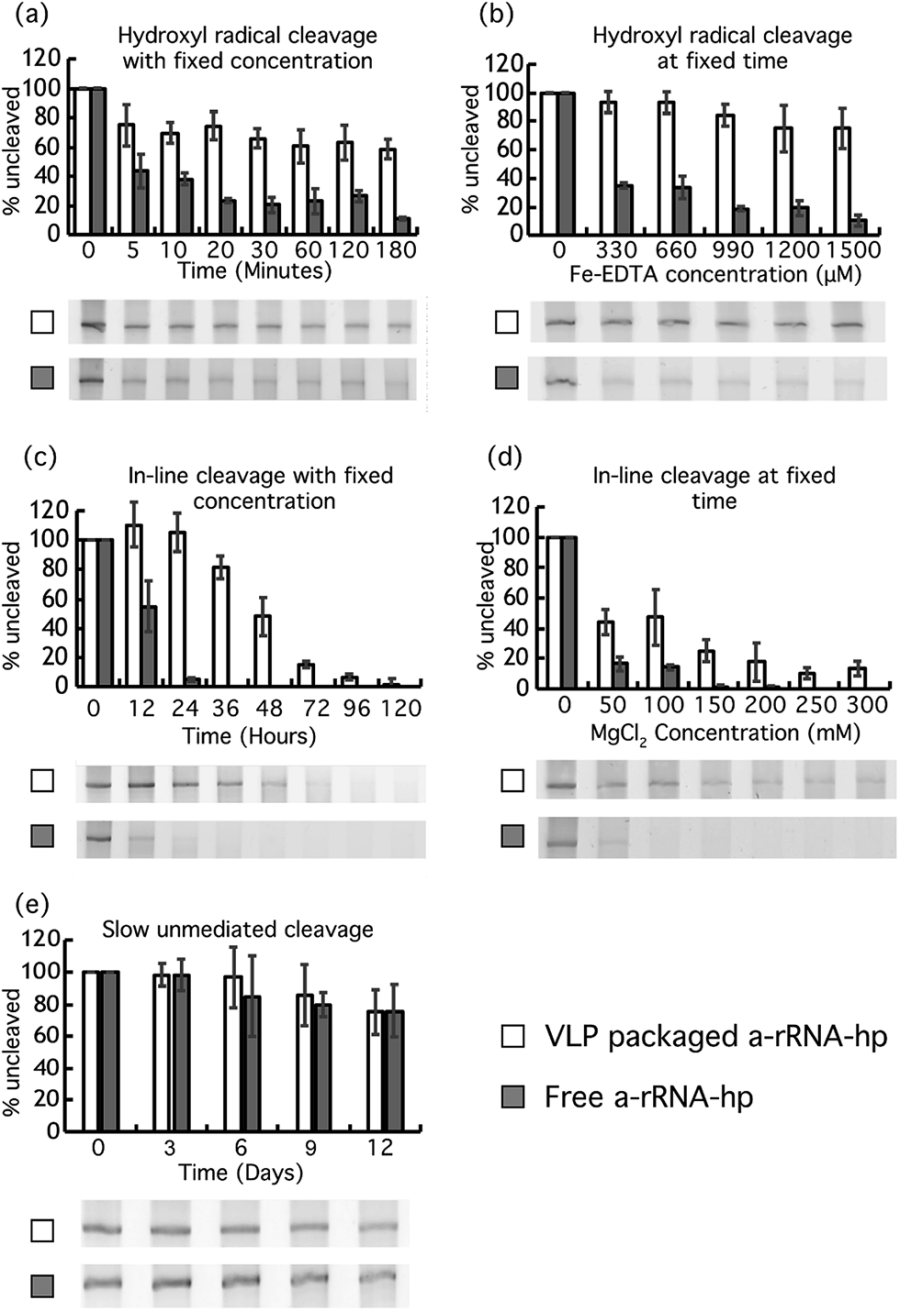
VLP packaging protects RNA. VLP–packaged a-rRNA-hp and free a-rRNA-hp were incubated under conditions (a) that promote Fenton chemistry (33 μM Fe-EDTA, H_2_O_2_ and atmospheric oxygen at 37 °C) at various timepoints, (b) that promote Fenton chemistry (varying [Fe-EDTA]) at constant time, (c) that promote inline cleavage (25 mM MgCl_2_ at 37 °C) at various timepoints, (d) that promote inline cleavage (varying [Mg^2+^] at 37 °C) at constant time, and (e) in the absence of divalent cations at neutral pH at various timepoints. For all experiments here, the uncleaved a-rRNA-hp was isolated on denaturing urea PAGE and quantitated with AlphaView® software. Error bars represent the 95% confidence interval of the quantitation.

Packaging in Qβ VLPs protects RNAs against attack by hydroxyl radical (Fig. 3a and b). We incubated VLPs with Fe-EDTA, O_2_ and H_2_O_2_, which are conditions known to generate hydroxyl radicals and to degrade RNA.^40^ Under the conditions of our time-dependent experiment, about 60% of a-rRNA-hp packaged within VLPs is intact after three hours, while less than 20% of the free RNA remains (Fig. 3a). The data suggest that hydroxyl radical cleaves RNA in a first order, bi-exponential process. Roughly three quarters of packaged RNA degrades slowly, while an equivalent proportion of free RNA is degraded quickly.

Similarly, protection of a-rRNA-hp within VLPs is observed in experiments in which the Fe-EDTA concentration is varied at fixed time (Fig. 3b). After 15 min in 1500 μM Fe-EDTA and 0.3% H_2_O_2_, about 80% of VLP–packaged a-rRNA-hp remains intact, while less than 20% of free RNA remains.

#### Magnesium-mediated in-line cleavage

Packaging protects a-rRNA-hp against magnesium-mediated in-line cleavage (Fig. 3c and d). In-line cleavage occurs through intramolecular attack by the nucleophilic 2′ hydroxyl on the proximal phosphate.

Magnesium is known to catalyze in-line cleavage of RNA.^37,41^ After 36 hours of incubation in 25 mM MgCl_2_ at 37 °C, the a-rRNA-hp within VLPs is about 80% intact. By contrast, less than 5% of free a-rRNA-hp is intact under the same conditions (Fig. 3c).

Similarly, VLP packaging increases the concentration of MgCl_2_ required for fixed extent of RNA cleavage at fixed time (Fig. 3d). About 45% of VLP–packaged a-rRNA-hp remains intact after 24 hours at a MgCl_2_ concentration of 100 mM. There is no detectable intact RNA remaining after the same incubation time for free RNA under the same conditions.

#### Unmediated cleavage

Finally, we assayed extent of unmediated cleavage of VLP–packaged RNA and free RNA (Fig. 3e). At neutral pH, RNA in the presence of buffer degrades very slowly at room temperature. Rates of unmediated cleavage are at least an order of magnitude less than rates of magnesium-mediated in-line cleavage or Fenton cleavage.

Our results show that both VLP–packaged and free a-rRNA-hp degrade slowly at 37 °C in the absence of Mg^2+^ and Fe^2+^-EDTA/H_2_O_2_. There is no significant difference in rates of degradation of VLP–packaged RNA and free RNA under the conditions of this experiment. The VLP does not appear to retard the rate of unmediated RNA degradation that occurs over extended periods of time.

### Effects of RNA length and intrinsic compaction on packaging in VLPs

**Fig. 4:**
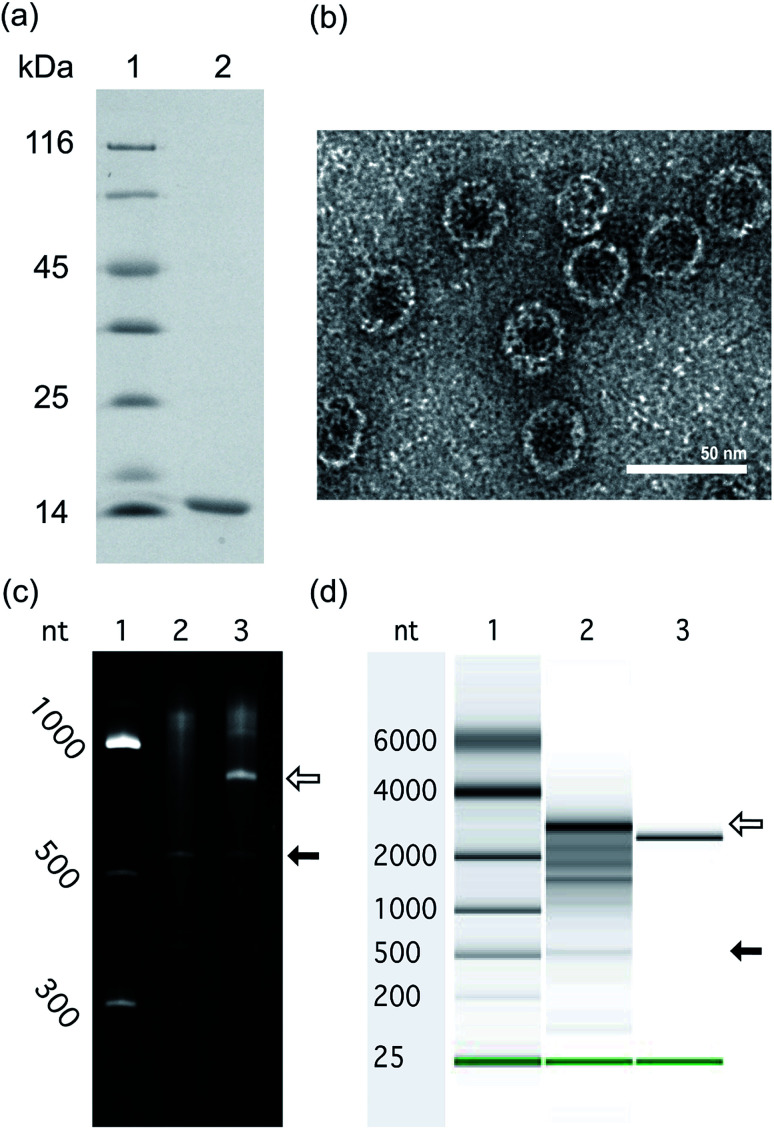
*In vivo* expressed RNA is packaged within VLPs to form VLP–RNA assemblies. (a) Purified Qβ VLPs analyzed by SDS-PAGE. The Qβ CP monomer is 14.5 kDa. Lane 1: protein sizing ladder. Lane 2: Qβ CP monomer from purified Qβ VLPs. (b) Transmission electron microscope images of Qβ VLP containing a-rRNA-hp. Scale bar = 50 nm. (c) *In vivo* expressed a-rRNA-hp, extracted from purified Qβ VLPs, subjected to electrophoresis on denaturing urea PAGE. Lane 1: RNA ladder, Lane 2: RNAs of Qβ CP expressed alone. Lane 3: RNAs of purified Qβ CP co-expressed with a-rRNA-hp. The open arrow indicates the a-rRNA-hp (747 nt). The closed arrow indicates the Qβ CP mRNA. (d) *In vivo* expressed 23S rRNA-hp, extracted from purified Qβ VLP, subjected to microfluidic-based chip electrophoresis. Lane 1: RNA sizing ladder, Lane 2: RNAs extracted from purified Qβ CP co-expressed with 23S rRNA-hp (3051 nt). Lane 3: 23S rRNA prepared by *in vitro* transcription (2966 nt). The open arrow in panel (d) indicates the 23S rRNA-hp. The closed arrow indicates the Qβ CP mRNA.

To investigate the impact of RNA size and structure on RNA packaging efficiency in Qβ VLPs, three representative RNAs of disparate length and structure were co-expressed with CP in *E. coli*. VLPs were purified and RNA packaging efficiency was estimated. SDS-PAGE analysis and transmission electron microscopy confirmed that co-expression of pCDF-CP and pET-RNA leads to expression of CP monomer (Fig. 4a) and canonical VLPs (Fig. 4b). RNA packaging was confirmed by denaturing urea PAGE of RNA extracted from purified VLPs (Fig. 4c and d).

#### Assay for packaging efficiency

**Fig. 5:**
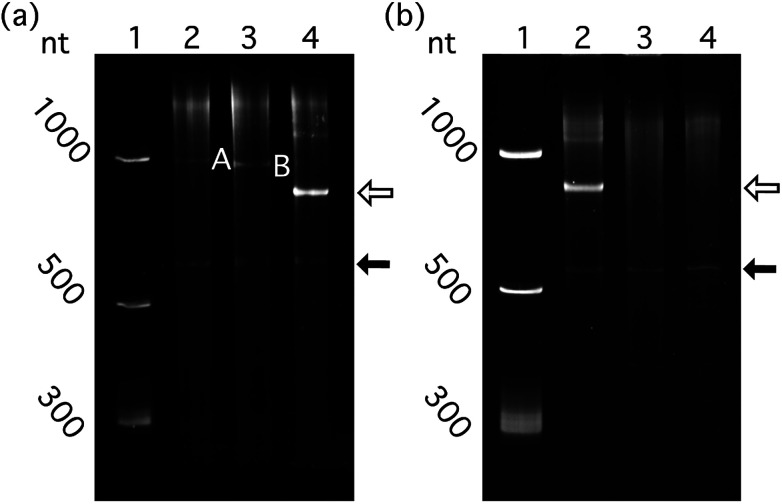
Highly structured RNAs bearing the Qβ hp and lacking the RBS package efficiently in Qβ VLP *in vivo*. (a) Structured and unstructured RNAs, packaged *in vivo* by Qβ CP. Lane 1: RNA ladder. Lane 2: RNAs extracted from purified Qβ CP co-expressed with RBS-mRNA_GFP_-hp. Lane 3: RNAs from purified Qβ CP co-expressed with mRNA_GFP_-hp. Lane 4: RNAs from purified Qβ CP co-expressed with a-rRNA-hp. (b) RNAs with and without the Qβ hp, co-expressed *in vivo* with Qβ CP. Lane 1: RNA ladder. Lane 2: RNAs of purified Qβ CP co-expressed with a-rRNA-hp. Lane 3: RNAs of purified Qβ CP co-expressed with a-rRNA (without Qβ hp). Lane 4: RNAs of Qβ CP expressed alone. One hundred nanograms of total VLP–extracted RNA was loaded to each well for denaturing PAGE. Band A: RBS-mRNA_GFP_-hp (851 nt). Band B: mRNA_GFP_-hp (833 nt, without the RBS). The open arrow indicates the a-rRNA-hp (747 nt). The closed arrow indicates the Qβ CP mRNA.

We used the amount of co-packaged CP mRNA as a proxy for packaging efficiency of target RNAs. VLPs contain mRNA encoding Qβ CP (Fig. 4c, d, and 5), as noted previously.^22^ Here we assume that the amount of CP mRNA packaged within VLPs is constant throughout this series of experiments. The amount of target RNA within the VLPs was quantitated and normalized relative to the amount of CP mRNA within the VLPs. Target RNAs packaged in great excess of CP mRNA are considered more efficiently packaged, while RNAs packaged at levels equivalent to CP mRNA are considered less efficiently packaged. Minor RNA impurities observed within VLPs appear to be endogenous *E. coli* RNA.

To probe the impact of RNA length and compactness on packaging efficiency, three appropriate RNAs were co-expressed with CP (Fig. 2).

#### RNA length

RNA length, over the range investigated here, does not appear to affect packaging efficiency within VLPs in *E. coli*. The results show, by the measure we use, that a-rRNA-hp (747 nt) and 23S rRNA-hp (3051 nt) are packaged with similar efficiencies (Fig. 4). The 23S rRNA-hp is roughly four times longer than a-rRNA-hp.

#### Intrinsic compaction

RNA compaction may play a role in packaging efficiency. It has been shown that a-rRNA^38^ and 23S rRNA^42,43^ fold to compact secondary and tertiary structures. We compared Qβ VLP packaging of mRNA_GFP_-hp to a-rRNA-hp and 23S rRNA-hp. The lengths of mRNA_GFP_-hp and a-rRNA-hp are similar. However, mRNA_GFP_-hp differs from a-rRNA-hp in that mRNA is less structured, less compact and more dynamic than rRNA. The results show that a-rRNA-hp is packaged more efficiently than mRNA_GFP_-hp (Fig. 5a), suggesting that highly structured RNA may be packaged more efficiently than less structured RNA.

We used gel mobility to qualitatively characterize the extent of compaction of a-rRNA-hp compared to mRNA_GFP_-hp. The mobility of a-rRNA-hp increases relative to mRNA_GFP_-hp when gel conditions are converted from non-native to native (data not shown). This increase in mobility is consistent with a greater compaction of a-rRNA-hp over mRNA_GFP_-hp in the native state.^44^

### RNA packaging efficiency is influenced by competing RNA binding factors

We determined whether ribosomes can compete with Qβ VLP packaging by binding target RNA. The effect of competition on packaging efficiency was assessed by co-expression of CP with pET-RNA encoding mRNA_GFP_-hp fused to the ribosomal binding site (RBS)^45^ (RBS-mRNA_GFP_-hp, Fig. 2b and f). Cloning at the XbaI/BlpI sites to generate all other constructs in the series of pET-RNAs used in this study eliminated the RBS of pET-28b (+). Therefore, mRNA_GFP_-hp represents a negative control for competition relative to RBS-mRNA_GFP_-hp.

Quantitative comparison of packaged RBS-mRNA_GFP_-hp with mRNA_GFP_-hp shows that RNA packaging efficiency in VLPs decreases when the RNA contains the RBS (Fig. 5a, *p* < 0.01, *n* = 6). This result suggests competition between ribosomal binding and Qβ assembly *in vivo*.

### Packaging of target RNA requires the Qβ hairpin

The Qβ hp is necessary for efficient packaging of essentially any RNA within a Qβ VLP. a-rRNA fused to the Qβ hp (a-rRNA-hp) shows the highest packaging efficiency of any RNA tested here. We investigated the importance of the Qβ hp to packaging of a highly structured RNA by removing Qβ hp from a-rRNA-hp (a-rRNA, Fig. 2b and h). Removal of the Qβ hp essentially abolishes packaging of a-rRNA (Fig. 5b).

Together, these results suggest that efficient packaging of RNA within VLPs *in vivo* may depend upon the compactness of the target RNA structure, the absence of competing binding motifs in the RNA, and the presence of the Qβ hp.

## Discussion

The potency and efficacy VLP–packaged RNA is related to the functionality, purity and quantity of the packaged RNA, and to the chemical stability of the RNA within the VLP. Using *E. coli* for combined expression and packaging of RNA into Qβ VLPs, we probed relationships between packaging efficiency and RNA size and intrinsic compaction. We characterized the degree of RNA protection from small diffusible agents such as hydroxyl radical and magnesium cations conferred by packaging in VLPs.

### RNA protection by VLP packaging

RNA packaged within MS2 VLPs is completely protected from digestion by plasma ribonucleases.^39^ The mechanism of this protection is steric; the enzymes are significantly larger than the pores in the VLP capsid. Here we establish that packaging in VLPs protects RNA against small, diffusible damaging agents that are smaller than the capsid pores.

We determine the level of protection afforded against metal ion mediated strand cleavage by packaging in Qβ VLPs. Mechanisms and levels of protection against small reactive molecules such as metal ions are expected to be distinct from those of nucleases because Qβ VLPs contain pores^20^ that are significantly larger than the metal ions and their hydrates. Mg^2+^ has an ionic diameter of 0.13 nm and a fully hydrated diameter of just less than 1 nm.^46^ Pores in VLPs allow entry and exit of metal ions and species such as hydroxyl radical. Diffusion of metal ions into Qβ VLPs has been demonstrated by Finn, who showed that RNA packaged in VLPs is degraded by lead(ii) acetate.^47^ It has been shown that RNA within MS2 VLPs is degraded by high pH.^48^

Our results demonstrate, as expected, that Mg^2+^ and Fe^2+^ enter VLPs and degrade RNA. The results are consistent with expectations that rates of RNA cleavage caused by these metals are highly attenuated for compact RNA packaged in the interior of VLPs compared to rates of cleavage of free RNA in solution. RNA is substantially protected against hydroxyl radical and magnesium-mediated in-line cleavage by packaging in VLPs (Fig. 3). It is known that rates of hydroxyl radical^49^ and magnesium-mediated in-line cleavage^41^ of RNA are influenced by RNA compaction and dynamics. RNA is more compact and less dynamic within VLPs than RNA that is free in solution.^50^

The RNA backbone is cleaved spontaneously in both the presence and in absence of magnesium.^37,41^ The mechanism is thought to be the same for both reactions; intramolecular in-line cleavage with a linear arrangement of the nucleophile (the 2′ oxygen), the electrophile (the P) and the leaving group (the 5′ oxygen). The reaction is faster in the presence of magnesium because it stabilizes the transition state by accepting a proton from the 2′ oxygen and/or by withdrawing electron density from the electrophilic phosphorous. Flexible regions of RNA are most labile to in-line cleavage because they more frequently occupy transient conformational states similar to the transition state.^37,41^ As expected, in the presence of magnesium the rate of cleavage decreases when RNA is packaged in the VLP. The reaction rate decreases because the dynamics of RNA within the VLP are suppressed by confinement and association with CP. Packaging of RNA in Qβ VLPs protects RNA not only against nucleases^51^ but against assault by small diffusible species.

### Structured RNA may pack more efficiently than unstructured RNA

The results indicate that *in vivo* RNA packaging of RNA within VLPs may be most efficient for intrinsically compact RNA such as rRNA and less efficient for dynamic, elongated RNA such as mRNA. The Qβ hp is necessary but not sufficient for efficient packaging.

Stockley previously suggested that for viral RNA, stem-loops dispersed through the MS2 viral genome interact with CP during assembly^52–54^ and tentatively identified 60 RNA stem-loops in the MS2 genome.^26^ RNA collapse during assembly *in vitro* depends initially on RNA–protein interactions, followed by CP–CP interactions.^55^ Packaging involves multiple weak RNA–CP interactions along the length of the viral RNA and a single strong interaction with the MS2 hp.^27^ MS2 and Qβ CP share about 25% amino acid sequence identity,^56–58^ with highly similar three-dimensional structures and RNA binding sites.

We conclude that *in vivo* packaging efficiency of non-viral RNA within Qβ VLPs may be high when the RNA (i) is intrinsically compact, (ii) includes the Qβ hp, (iii) lacks binding sites for assemblies that compete with CP (*e.g.*, the RBS), and (vi) is as large as the 23S rRNA. We prepared a series of RNAs (Fig. 2) and co-expressed the RNAs with Qβ CP in *E. coli*. The RNAs expressed here contain (i) a-rRNA, a ∼650 nt intrinsically compact RNA stabilized by 11 GNRA tetraloops,^38^ (ii) 23S rRNA, a ∼2970 nt intrinsically compact RNA containing over forty stem-loops, or (iii) mRNA_GFP_, an unstructured ∼780 nt RNA. The expressed RNAs either contained or lacked the Qβ hp, and/or a RBS. The results show that both a-rRNA-hp and 23S rRNA-hp are efficiently packaged within Qβ VLP *in vivo* (Fig. 4), suggesting that, within the limits explored here, RNA length is not a strong predictor of packaging efficiency. We compared the packaging efficiency of intrinsically compact a-rRNA-hp and unstructured mRNA_GFP_-hp. The results of these experiments show greater packaging of intrinsically compact rRNA than unstructured mRNA (Fig. 5a). We conclude that intrinsic RNA compaction influences the efficiency of *in vivo* RNA packaging in Qβ VLPs.

### Qβ hp is indispensable for efficient packaging of RNA

The Qβ hp is necessary but not sufficient for efficient packaging of RNA into VLPs within *E. coli* (Fig. 5b). This finding is consistent with previous work showing that RNA lacking the MS2 hairpin does not interact with MS2 CP *in vitro*.^55^ The MS2 hairpin is thought to be the initiation trigger for viral capsid formation.^59–62^ Our results confirm that numerous GNRA tetraloops found on the surface of a-rRNA,^38^ do not substitute for the Qβ hp during RNA packaging *in vivo*.

### Competing binding processes attenuate RNA packaging in VLPs

Finn and coworkers found that VLP packaging of CP mRNA competes with CP expression. When the Qβ hp is fused to the 3′ end of the CP mRNA, expression of Qβ CP decreases.^63^ Our results show that target RNA packaging efficiency in VLPs is slightly decreased when an RBS is introduced upstream of the mRNA_GFP_-hp (Fig. 5a). The presence of the RBS decreases RBS-mRNA_GFP_-hp packaging in the VLP relative to mRNA_GFP_-hp, suggesting that Qβ CP and the ribosome compete for binding to RBS-mRNA_GFP_-hp. Based on previous studies and our current findings, we expect that introduction of additional high-affinity RNA binding sites could facilitate or attenuate RNA packaging efficiency.

### Off-target RNA packaging

We and others observe that non-target RNAs, including Qβ CP mRNA, tRNAs, *etc.*, are packaged within VLPs along with target RNA. Contaminating RNAs compete with target RNA during co-assembly. The absolute amount of contaminating RNA appears to depend of the extent of packaging of the target RNA. If target RNA is expressed at low levels, contains motifs (*e.g.*, RBS) with affinity for competing species or lacks the Qβ hp, then the amount of contaminating RNA is increased. In total, it appears that sufficient RNA is packaged in the VLP to fully compensate for the cationic charge of Qβ CP.^64^ It is possible that RNA is required within the VLP for neutralization of positive charge.^65^ In our two-plasmid expression system, T7 RNA polymerase was used to induce high-level expression of both Qβ CP and target RNA. Without the simultaneous expression of RNA *in vivo*, high concentrations of positively charged viral CP may form non-specific RNA–CP interactions that serve to stabilize the protein structure and neutralize RNA charge.^66^

### Relevance to VLP-based therapies

Qβ VLPs are non-infectious and non-replicative platforms for delivery of various therapies with high stability and biocompatibility.^67^ VLPs have been shown to enhance the immunogenicity of some peptides through surface display.^68^ Expression of Qβ VLPs in *E. coli* packages host RNAs capable of inducing immune response in animal models, an incidental adjuvant.^67,69^ Several VLP-based therapies have already shown “good safety and tolerability” in clinical trials.^67^

The immunogenicity of VLPs presents a challenge for their use in systematic delivery. However, efforts to attenuate immunogenicity are in progress in several laboratories, for example by “PEGylation”. This approach has been shown to limit the primary immune response of mice to cowpea mosaic virus,^70^ and to decrease immune response in rats administered adenovirus-based therapy targeting metastatic colonies.^71^ PEG is already clinically approved and is in use in pharmaceuticals.

We previously demonstrated delivery of functional RNAi to cancer cell lines using a highly structured RNAi scaffold co-expressed with VLPs in *E. coli*.^15^ The current study explored the role of RNA sequence and structure in efficient packaging by co-expression in *E. coli*, and the stability of RNA within VLPs once purified. Though these specific RNAs are not intended for use in VLP-based therapies, they represent a range of properties relevant to the development of RNA-based VLP therapeutics.

## Conclusions

Using *E. coli* for combined expression and packaging of non-viral RNA in Qβ VLPs, we probed relationships between *in vivo* packaging efficiency and RNA size, sequence, and intrinsic compaction. We purified assembled VLPs and evaluated the extent of resistance to metal ion mediated degradation conferred on RNA by packaging in VLPs. We conclude that *in vivo* packaging in Qβ VLPs (i) protects target RNA against cleavage by hydroxyl radical and magnesium, and (ii) is efficient when the target RNA is intrinsically compact, includes the Qβ hairpin, and lacks the RBS.

## Conflicts of interest

There are no conflicts to declare.

## Supplementary Material

RA-008-C8RA02084A-s001
