# Supplementary material for: RNA: packaged and protected by VLPs
Source: RSC Adv. 2018 Jun 12;8(38):21399–406. doi: 10.1039/c8ra02084a (PMC9080931; doi:10.1039/c8ra02084a)
Supplement: RA-008-C8RA02084A-s001 [file RA-008-C8RA02084A-s001.pdf]

Electronic Supplementary Material (ESI) for RSC Advances.
This journal is © The Royal Society of Chemistry 2018

Supporting Information

RNA: Packaged and Protected by VLPs

Po-Yu Fang,[a] *Jessica C. Bowman,*[a] *Lizzette M. Gómez Ramos,*[a,b] *Chiaolong Hsiao,*[c] *and Loren Dean Williams*[a,*]

[a]School of Chemistry and Biochemistry, Georgia Institute of Technology, Atlanta, GA, 30332, USA, [b]School of Chemical and Biomolecular Engineering, Georgia Institute of Technology, Atlanta, GA 30332, USA, [c] Institute of Biochemical Sciences, National Taiwan University, Taipei 10617, Taiwan, R.O.C.

* Address correspondence to Loren Dean Williams.

Reagents. DNA oligomers were obtained from Eurofins MWG Operon, Inc. All DNA constructs were confirmed by sequencing (Eurofins MWG Operon). NZY broth media was purchased from TEKNOVA. Sec-Butanol and Chloroform and Magnesium Chloride hexahydrate were purchased from Fisher Scientific. Ferrous Ammonium Sulfate Hexahydrate was obtained from EM Science. Ammonium sulfate was purchased from ICN Biomedicals. Sucrose, RNase- and DNase-free, was purchased from Amresco. Dithiothreitol (DTT) were purchased from Research Products International Corp. (+)-sodium L-ascorbate was purchased from Sigma-Aldrich. Sodium dodecyl sulfate was purchased from Shelton Scientific, Inc. Polyethylene glycol 8000 was purchased from J. T. Baker. 30% hydrogen peroxide was purchased from Fisher Scientific. BCA protein assay kit was purchased from Thermo Scientific. Low range ssRNA ladder was purchased from New England Biolabs. Ultra-Pure SequGel was purchased from National Diagnostics. Polyallomer centrifuge tubes were purchased from Beckman Coulter. Agilent RNA 6000 Nano Kit was purchased from Agilent Technologies. Amicon® Ultra centrifugal filters (100 kDa MWCO) were purchased from Millipore. Spectra/Por® dialysis tubing (15 KDa MWCO) was purchased from Spectrum® Laboratories, Inc. All other reagents were analytical grade.

Table S1. Primers and oligomers used for construction of the Qβ CP gene[a]

Oligomer name	DNA sequence[b]
CP-Fwd[c]	5'- GTG G<u>CC ATG G</u>CA AAT TAG AGA CTG TTA CTT -3'
CP-Rev[d]	5'- CAC C<u>CC TAG G</u>TC AAT ACG CTG GGT TC -3'

QβCP-F1	5’- GTG GGC TCA GCT CAA TAC GCT GGG TTC AGC TGA TCA ATA GCA TCG ATC AGC AGA GGA CTA -3’
QβCP-R1	5’- GCT TTT GTT CGT ACA GAG CTT GCT GCT CTG CTC GCT AGT CCT CTG CTG ATC GAT GCT ATT -3’
QβCP-F2	5’- GCA AGC TCT GTA CGA ACA AAA GCT CGT TCC TCA TCG GTA CTA TAC TGC GTG AAC GAA AAG GTC -3’
QβCP-R2	5’- TTG TGA CCC ATC CGT TAC TCG CCA GGC ATA TGC TGA CGT GAC CTT TTC GTT CAC GCA GTA TAG TA -3’
QβCP-F3	5’- CGA GTA ACG GAT GGG TCA CAA GAA CCG TTT GCA GTG CAA GCG GTC GGG TTC TGG ATC TTA ACC TGG ACC -3’
QβCP-R3	5’- CCG TTT CGG TAT CTC AGC CTT CTC GCA ATC GTA AGA ACT ACA AGG TCC AGG TTA AGA TCC AGA ACC -3’
QβCP-F4	5’- AAG GCT GAG ATA CCG AAA CGG TAA CAC GCT TCT CCA GCG CAG GAA CTG CAC CCG CTT GTG AAA GCG-3’
QβCP-R4	5’- TCT GGT CCT CAA TCC GCG TGG GGT AAA TCC CAC TAA CGG CGT TGC CTC GCT TTC ACA AGC GGG TG -3’
QβCP-F5	5’- ACG CGG ATT GAG GAC CAG AGT TTG TTT TCC ATC TTT CCC GAT GTT ACC TAA AGT AAC AGT C -3’
QβCP-R5	5’- CAC CCC ATG GGC AAA ATT AGA GAC TGT TAC TTT AGG TAA CAT CGG GAA -3’

a) Genes were constructed by recursive PCR (Bowman et al. 2012).

b) Restriction sites are underlined.

c) CP-Fwd is the forward primer, which contains an *Nco*I site.

d) CP-Rev is the reverse primer, which contains an *Avr*II site.

Table S2. Primers and oligomers used for non-viral RNA gene construction.

**a-rRNA-hp**[a,b,c,d,e]	**Primer or oligomer sequence**
a-rRNA-hp FWD	5’- GTG GTC TAG A*GT CCG AGT AAT TTA CGT TTT GA* -3’
a-rRNA-hp REV	5’- GGT GGC TCA GCG CGA AGA TGC TGT -3’

a-rRNA-hp oligo F1	5’- TCT AGA *GTC CGA GTA ATT TAC GTT TTG ATA CGG TTG CGG AAC TTG C*GG GGT GCC TAT TGA AGC ATG -3’
a-rRNA-hp oligo F2	5’- TCT CTA TCC GCC ACG GGC TTC CTC GTG CTT AGT AAC TAA GGA TGA AAT GCA TGT C -3’
a-rRNA-hp oligo R2	5’- GCT CAG CGC GAA GAT GCT GTC TTA GAC ATG CAT TTC ATC CTT AGT TAC TAA GC -3’

23S rRNA-hp hp[a,b,c,d,e]	Primer or oligomer sequence
23S rRNA-hp FWD	5’- GTG GTC TAG A*GT CCG AGT AAT TTA CGT TTT GA* -3’
23S rRNA-hp REV	5’- GGT GGC TCA GCG CGA AGA TGC TGT -3’
23S rRNA-hp oligo F1	5’-TCT AGA *GTC CGA GTA ATT TAC GTT TTG ATA CGG TTG CGG AAC TTG C* -3’
23S rRNA-hp oligo R1	5’- GCA TCC ACC GTG GGC CCT TAC CAT CTT GAC *GCA AGT TCC GC AAC CGT AT C* -3’
23S rRNA-hp oligo F2	5’- CCG AGG TCT TGA CCC CTC CTT CCT CGT GCT TAG TAA CTA AGG ATG AAA TG -3’
23S rRNA-hp oligo R2	5’- GCT CAG CGC GAA GAT GCT GTC TTA GAC ATG CAT TTC ATC CTT AGT TAC TAA GCA CGA G -3’

$mRNA_{GFP}$-hp [d,e]	Primer or oligomer sequence
$mRNA_{GFP}$-hp FWD	5’- GTG GTC TAG AAT GGC TAG CAA AGG AGA AGA ACT CT -3’
$mRNA_{GFP}$-hp REV	5’- GGT GGC TCA GCG CGA AGA TGC TGT -3’

a) The Qβ hp is highlighted in blue.

**RBS-mRNA$_{GFP}$- hp[a,b,c]**	**Primer or oligomer sequence**
RBS-mRNA$_{GFP}$-hp FWD	5'- GTG GTC TAG A AT GGC TAG CAA AGG AGA AGA ACT CT -3'
RBS-mRNA$_{GFP}$-hp REV	5'- GGT GGC TCA GCG CGA AGA TGC TGT -3'
**RBS-mRNA$_{GFP}$-hp oligo F1**	5'- TCT AGA ATA ATT TTG TTT AAC TTT AAG AAG GAG ATA TAC CAT GGC TAG CAA AGG AGA AGA ACT CT -3'

**a-rRNA[a,b,d]**	**Primer or oligomer sequence**
**a-rRNA FWD**	5'- GTG GTC TAG A*GT CCG AGT AAT TTA CGT TTT GA* -3'
**a-rRNA REV**	5'- GGT GGC TCA GCG CCC GTG GCG GAT AGA GA -3'

b) The spacer region highlighted in pink *italics*.

c) The ribosomal binding site (RBS), highlighted in red.

d) Overlapping recombinant RNA fragments, including a-rRNA, 23S rRNA, and GFP mRNA are highlighted in yellow.

e) *Xba*I/*Blp*I sites underlined.

References:

Bowman JC, Azizi B, Lenz TK, Roy P, Williams LD. 2012. Preparation of long templates for RNA in vitro transcription by Recursive PCR. In *Recombinant and In Vitro RNA Synthesis: Methods and Protocols, Methods in Molecular Biology*, Vol 941 (ed. GL Conn), pp. 19-41. Springer Science, LLC.
